# Impact of the Great East Japan Earthquake on Body Mass Index, Weight, and Height of Infants and Toddlers: An Infant Survey

**DOI:** 10.2188/jea.JE20170006

**Published:** 2018-05-05

**Authors:** Hiroshi Yokomichi, Hiroko Matsubara, Mami Ishikuro, Masahiro Kikuya, Tsuyoshi Isojima, Susumu Yokoya, Noriko Kato, Toshiaki Tanaka, Shoichi Chida, Atsushi Ono, Mitsuaki Hosoya, Soichiro Tanaka, Shinichi Kuriyama, Shigeo Kure, Zentaro Yamagata

**Affiliations:** 1 Department of Health Sciences, University of Yamanashi, Chuo, Yamanashi, Japan; 2 Department of Disaster Public Health, International Research Institute of Disaster Science, Tohoku University, Sendai, Japan; 3 Tohoku Medical Megabank Organization, Tohoku University, Sendai, Japan; 4 Department of Molecular Epidemiology, Tohoku University, Sendai, Japan; 5 Department of Paediatrics, Teikyo University, Tokyo, Japan; 6 Center for Clinical Research Data, National Center for Child Health and Development, Tokyo, Japan; 7 Department of Early Childhood Care and Education, Jumonji University, Niiza, Saitama, Japan; 8 Japanese Association for Human Auxology, Tokyo, Japan; 9 Department of Paediatrics, Iwate Medical University, Morioka, Japan; 10 Department of Paediatrics, Fukushima Medical University, Fukushima, Japan; 11 Department of Paediatrics, Tohoku University, Sendai, Japan

**Keywords:** body mass index, infant, growth, earthquake, Fukushima nuclear accident

## Abstract

**Background:**

The body mass index (BMI) of preschool children from 4 years of age through primary school has increased since the Great East Japan Earthquake, but that of children aged under 3 years has not been studied. This study evaluated how the anthropometrics of younger children changed following the earthquake.

**Methods:**

Height and weight data of children living in northeast Japan were collected from 3-, 6-, 18-, and 42-month child health examinations. We compared the changes in BMI, weight, and height among infants affected by the earthquake between their 3- and 6-month health examinations, toddlers affected at 21–30 months of age (affected groups), and children who experienced the earthquake after their 42-month child health examination (unaffected group). A multilevel model was used to calculate the BMI at corresponding ages and to adjust for the actual age at the 3-month health examination, health examination interval, and gestational age.

**Results:**

We recruited 8,479 boys and 8,218 girls living in Fukushima, Miyagi, and Iwate Prefectures. In the infants affected between their 3- and 6-month health examinations in Fukushima, the change in BMI at 42 months of age was greater than among the unaffected children. In the toddlers affected at 21–30 months of age in Fukushima, the change in BMI was greater, but changes in weight and height were less.

**Conclusions:**

Affected infants and toddlers in Fukushima suggested some growth disturbances and early adiposity rebound, which can cause obesity. The future growth of children affected by disasters should be followed carefully.

## INTRODUCTION

The Great East Japan Earthquake registered 9.0 on the moment magnitude scale and was the largest in Japan since 1875.^1^ It occurred on March 11, 2011 on the Pacific coast of northeast Japan, damaged a substantial number of homes, buildings,^2^ and schools, and reportedly resulted in health problems for many residents.^3^^–^^7^ There have, for example, been reports of increasing body mass index (BMI) in preschool children of about 4 years of age.^8^^–^^10^ Problems in Fukushima Prefecture, where a tsunami following the earthquake destroyed a nuclear power station, were prolonged.

Following an earthquake, micronutrient deficiency in children during the acute phase^11^ and growth stunting in the post-quake phase^12^ is usually a problem. Studies conducted after the Great East Japan Earthquake show that dietary sources were restricted during the acute phase,^13^ and outdoor physical activity decreased during the post-quake phase because of fear of exposure to radioactive discharge from damaged nuclear power plants.^14^ Fear of death may change hormone levels among those affected.^15^ Experts suggest that growth retardation during the first 2–3 years of life can cause undesirable health outcomes later in childhood.^16^^,^^17^ Studies on the impact of the earthquake have been limited to preschool children about 4 years of age,^8^ and little is known about the influence on children younger than 3 years of age. The aim of this study was to determine: 1) whether the growth of infants and toddlers was disturbed by the earthquake; and 2) how the earthquake changed the BMI of children living on the Pacific coast of northeast Japan.

## METHODS

### Participants and measurements

With municipal government assistance, we invited children living along the Pacific coast of northeast Japan (ie, Fukushima, Miyagi, and Iwate Prefectures) to participate in a survey on infant growth. The details of the Health Examination Survey on Early Childhood Physical Growth in the Great East Japan Earthquake Affected Areas have been published elsewhere.^18^ In total, 31 out of 59 municipalities (52.5%) in Fukushima Prefecture, 19 out of 36 (54.3%) in Miyagi Prefecture, and 30 out of 33 (90.9%) in Iwate Prefecture collaborated with this survey and provided data from 3-, 6-, 18-, and 42-month child health exams. During the healthcare visits, the children’s gestational age and birth weight were collected through an interview or from a maternal and child health handbook, and their weight and height were measured. We compared the BMIs of three study groups: the ‘group affected between 3 and 6 months old’ included infants who experienced the earthquake between their 3- and 6-month health exams; the ‘group affected between 21 and 30 months old’ included children between 21–30 months of age at the earthquake and between their 18- and 42-month health exams; and the ‘unaffected group’ (reference group for comparison) included any child who had completed a 42-month health exam before the earthquake. This last group’s anthropometric data were not considered to have been affected by the earthquake. Through the municipalities, we invited 20,600 children from Fukushima, 15,804 from Miyagi, and 17,932 from Iwate who were born from March 2007 through April 2011. At the baseline of the 3-month health exam, 8,855 boys and 8,508 girls in Fukushima, 4,293 boys and 4,041 girls in Miyagi, and 6,851 boys and 6,714 girls in Iwate were included. The data with analyzable values included 2,890 children from Fukushima, 461 from Miyagi, and 2,309 from Iwate in the group affected between 3 and 6 months old; 2,331, 422, and 1,508 in the group affected between 21 and 30 months old; and 3,212, 1,190, and 2,374 in the unaffected group, respectively. These figures constitute around 13.9% of children in Fukushima, 2.6% in Miyagi, and 15.2% in Iwate, according to the numbers of children enrolling in primary school in the following years.^19^

The primary outcome in the group affected between 3 and 6 months old was the change in BMI (weight in kilograms divided by the square of height in meters) from a baseline age of 3 months to 42 months old compared with the unaffected group. The primary outcome in the group affected between 21 and 30 months old was the change in BMI from a baseline age of 18 months to 42 months old compared with the unaffected group. The secondary outcomes in the two affected groups were the changes in weight and height compared with the unaffected group. The data of the children who received each of the 3-, 6-, 18-, and 42-month health exams were analyzed for the group affected between 3 and 6 months old. The data of those who received both the 18- and 42-month health exams were analyzed for the group affected between 21 and 30 months old.

### Statistical analysis

To determine the influence of the earthquake on continuously growing children, we estimated and compared the changes in the anthropometrics from 3 to 6, 18, and 42 months of age between the children affected between their 3- and 6-month health exams and the unaffected children. We also compared the changes from 18 to 42 months of age between the children affected at between 21 and 30 months old and the unaffected children. We used a multilevel model (general linear model) to adjust for the gestational age, actual age at the 3-month health exam, and the intervals between the 3-month and subsequent exams. Bonferroni’s method^20^ was used to adjust for the level of statistical significance, correcting for comparisons at three time points between the groups affected between 3 and 6 months old and not affected. The *P*-values were evaluated at one-third of the level of the usual statistical significance. For instance, we applied a singular multilevel model to estimate the changes in the BMI from the age of 3 to 6, 18, and 42 months in the children affected between their 3- and 6-month exams and the unaffected children by strata of prefecture and sex. In the same model equation, we estimated the change in BMI from the age of 18 to 42 months in the children affected at between 21 and 30 months old. The estimated coefficients were used to calculate the adjusted changes in BMI at an actual age of 6, 18, and 42 months. A total of 12 models were used for the boys and girls of Fukushima, Miyagi, and Iwate Prefectures. The model for the change in BMI was:

$$\begin{array}{l} (\text{change in BMI at the 6-, 18- or 42-month exam}{)}_{\text{ij}}\\ \;=\beta_{0}+\beta_{\text{1}}(\text{group}{)}_{\text{i}}\text{+}\beta_{\text{2}}(\text{actual age at the 3-month exam}{)}_{\text{ij}}\\ \;+\beta_{\text{3}}(\text{interval between the 3- and corresponding 6-, 18-},\\ \text{ or 42-month exam}{)}_{\text{ij}}\\ \;+\beta_{\text{4}}(\text{gestational age}{)}_{\text{ij}}\text{+}\varepsilon_{\text{ij}}\text{ and }\varepsilon_{\text{ij}}\sim \text{N}(\text{0},\sigma^{\text{2}}), \end{array}$$

where i represents the indices of the unaffected or affected groups between the 3- and 6-month exams, and j represents the indices of the individual infants. Betas are regression coefficients and ε_ij_ is the residual term in the model. We plotted the mean adjusted change in BMI at 6, 18, and 42 months of age as calculated using the model coefficients.

For sensitivity analyses, changes in BMI were estimated using a similar model, with adjustment of the birth weight or BMI at the 3-month health exam, instead of the gestational age. In the plotted figure, the statistical significance of the differences between each affected group and the unaffected group was assessed using the *P*-value of the coefficients (β_1_). All statistical analyses used SAS statistical software (version 9.3, SAS Institute, Cary, NC, USA). Descriptive statistics were reported as the means and standard deviations (SD). All reported *P*-values were two-sided; *P*-values <0.05 were considered to be statistically significant.

### Ethical considerations

The ethics committee of the Tohoku University School of Medicine approved the study protocol (no. 2012-1-125). The study was conducted following the ethical guidelines and regulations of the Declaration of Helsinki. The data were collected anonymously, and the participants could choose to opt out of the study. This study was an analysis of the data of the municipal healthcare service, and informed consent was therefore not required for this investigation according to the Japanese guidelines.

## RESULTS

### Analyzed children

Table 1 shows the mean birth weight and gestational age of the participating infants from Fukushima, Miyagi, and Iwate Prefectures. The gestational age and birth weight were similar to those of neonates in the general population of Japan.^21^ Table 2 shows the mean BMI, weight, and height of the study population at 3, 6, 18, and 42 months of age by prefecture, sex, and group.

**Table 1.** Birth weight and gestational age of children living in Fukushima, Miyagi, and Iwate Prefectures

Mean (standard deviation)	Unaffected	Affected at 3–6 mo	Affected at 21–30 mo	Mean (standard deviation)	Unaffected	Affected at 3–6 mo	Affected at 21–30 mo
**Fukushima, boys, *n***	***n* = 1,646**	***n* = 1,479**	***n* = 1,166**	**Fukushima, girls, *n***	***n* = 1,566**	***n* = 1,411**	***n* = 1,165**
Birth weight, g	3,052 (423)	3,049 (402)	3,078 (415)	Birth weight, g	2,989 (420)	2,969 (387)	2,975 (416)
Gestational age, weeks	39.2 (1.5)	39.4^*^ (1.5)	39.4^*^ (1.5)	Gestational age, weeks	39.4 (1.6)	39.5 (1.8)	39.5 (1.6)

**Miyagi, boys, *n***	***n* = 602**	***n* = 232**	***n* = 219**	**Miyagi, girls, *n***	***n* = 588**	***n* = 229**	***n* = 203**
Birth weight, g	3,087 (420)	3,100 (397)	3,067 (410)	Birth weight, g	2,994 (402)	2,950 (471)	3,001 (364)
Gestational age, weeks	39.1 (1.6)	39.1 (1.4)	39.1 (1.6)	Gestational age, weeks	39.4 (1.5)	39.4 (2.2)	39.2 (1.4)

**Iwate, boys, *n***	***n* = 1,209**	***n* = 1,176**	***n* = 750**	**Iwate, girls, *n***	***n* = 1,165**	***n* = 1,133**	***n* = 758**
Birth weight, g	3,045 (424)	3,047 (423)	3,051 (415)	Birth weight, g	2,988 (392)	2,962 (412)	2,959 (412)
Gestational age, weeks	39.4 (1.6)	39.4 (1.5)	39.4 (1.6)	Gestational age, weeks	39.6 (1.5)	39.5 (1.6)	39.5 (1.6)

Data show the means (standard deviation). ^*^ indicates statistical significance at a *P* level <0.05 vs. the unaffected group.

**Table 2.** Actual BMI, weight, and height of infants and toddlers in Fukushima, Miyagi, and Iwate Prefectures before and after the Great East Japan Earthquake

**Boys in Fukushima**	**Unaffected**	**Affected at 3–6 mo**	**Affected at 21–30 mo**	**Girls in Fukushima**	**Unaffected**	**Affected at 3–6 mo**	**Affected at 21–30 mo**
Number of participants	1,646	1,479	1,166		1,566	1,411	1,165
**BMI, kg/m^2^**				**BMI, kg/m^2^**			
at 3 mo	17.64 (1.54)	17.53^*^ (1.52)	17.57 (1.50)	at 3 mo	17.18 (1.37)	17.15 (1.40)	17.16 (1.50)
at 6 mo	17.37 (1.35)	17.26^*^ (1.39)	17.42 (1.36)	at 6 mo	17.07 (1.33)	16.95^*^ (1.34)	17.11 (1.38)
at 18 mo	16.37 (1.15)	16.41 (1.17)	16.31 (1.16)	at 18 mo	16.03 (1.18)	16.07 (1.12)	15.93^*^ (1.21)
at 42 mo	15.76 (1.19)	15.89^*^ (1.11)	15.78 (1.17)	at 42 mo	15.65 (1.21)	15.78^*^ (1.18)	15.65 (1.24)
**Weight, g**				**Weight, g**			
at 3 mo	7,227 (800)	7,181 (804)	7,249 (774)	at 3 mo	6,734 (731)	6,693 (717)	6,746 (774)
at 6 mo	9,092 (910)	9,041 (956)	9,078 (909)	at 6 mo	8,561 (875)	8,554 (889)	8,572 (926)
at 18 mo	10,836 (1,061)	10,804 (1,072)	10,780 (1,069)	at 18 mo	10,303 (1,057)	10,287 (1,021)	10,217^*^ (1,075)
at 42 mo	14,555 (1,661)	14,503 (1,554)	14,448 (1,603)	at 42 mo	14,204 (1,672)	14,150 (1,591)	14,052^*^ (1,667)
**Height, cm**				**Height, cm**			
at 3 mo	64.0 (2.2)	64.0 (2.2)	64.2^*^ (2.2)	at 3 mo	62.6 (2.2)	62.4 (2.1)	62.6 (2.3)
at 6 mo	72.3 (2.5)	72.3 (2.5)	72.2 (2.4)	at 6 mo	70.8 (2.5)	71.0^*^ (2.4)	70.7 (2.6)
at 18 mo	81.3 (2.7)	81.1^*^ (2.7)	81.2 (2.7)	at 18 mo	80.1 (2.8)	80.0 (2.7)	80.0 (2.7)
at 42 mo	96.0 (2.9)	95.4^*^ (3.4)	95.6^*^ (3.5)	at 42 mo	95.2 (3.6)	94.6^*^ (3.5)	94.6^*^ (3.5)

**Boys in Miyagi**	**Unaffected**	**Affected at 3–6 mo**	**Affected at 21–30 mo**	**Girls in Miyagi**	**Unaffected**	**Affected at 3–6 mo**	**Affected at 21–30 mo**
Number of participants	602	232	219		588	229	203
**BMI, kg/m^2^**				**BMI, kg/m^2^**			
at 3 mo	17.82 (1.65)	18.04 (1.62)	17.71 (1.42)	at 3 mo	17.18 (1.47)	17.34 (1.47)	17.41 (1.56)
at 6 mo	17.68 (1.51)	17.36^*^ (1.42)	17.64 (1.34)	at 6 mo	17.23 (1.39)	16.81^*^ (1.44)	17.38 (1.41)
at 18 mo	16.58 (1.30)	16.49 (1.17)	16.48 (1.13)	at 18 mo	16.11 (1.20)	16.07 (1.22)	16.18 (1.21)
at 42 mo	15.95 (1.29)	16.04 (1.16)	15.89 (1.08)	at 42 mo	15.78 (1.26)	15.81 (1.19)	15.86 (1.44)
**Weight, g**				**Weight, g**			
at 3 mo	7,250 (835)	7,171 (811)	7,241 (842)	at 3 mo	6,643 (775)	6,615 (731)	6,814^*^ (788)
at 6 mo	9,034 (1,246)	9,075 (1,064)	8,800^*^ (1,096)	at 6 mo	8,412 (1,144)	8,466 (947)	8,308 (1,024)
at 18 mo	11,163 (1,235)	10,994^*^ (1,059)	10,852^*^ (1,040)	at 18 mo	10,480 (1,023)	10,366 (1,053)	10,427 (1,091)
at 42 mo	15,095 (1,859)	15,055 (1,606)	14,867 (1,616)	at 42 mo	14,563 (1,676)	14,574 (1,661)	14,665 (1,953)
**Height, cm**				**Height, cm**			
at 3 mo	63.8 (2.3)	63.0^*^ (2.5)	63.9 (2.5)	at 3 mo	62.1 (2.5)	61.7^*^ (2.1)	62.5 (2.3)
at 6 mo	71.4 (4.2)	72.3^*^ (3.8)	70.6^*^ (3.8)	at 6 mo	69.8 (4.2)	71.0^*^ (3.4)	69.1^*^ (3.6)
at 18 mo	82.0 (2.7)	81.6 (2.8)	81.1^*^ (2.5)	at 18 mo	80.6 (2.7)	80.3 (2.4)	80.2 (2.7)
at 42 mo	97.1 (3.5)	96.8 (3.5)	96.6^*^ (3.4)	at 42 mo	96.0 (3.3)	95.9 (3.5)	96.0 (3.8)

**Boys in Iwate**	**Unaffected**	**Affected at 3–6 mo**	**Affected at 21–30 mo**	**Girls in Iwate**	**Unaffected**	**Affected at 3–6 mo**	**Affected at 21–30 mo**
Number of participants	1,209	1,176	750		1,165	1,133	758
**BMI, kg/m^2^**				**BMI, kg/m^2^**			
at 3 mo	17.49 (1.57)	17.46 (1.51)	17.48 (1.46)	at 3 mo	17.04 (1.51)	17.03 (1.53)	17.05 (1.44)
at 6 mo	17.49 (1.40)	17.39 (1.38)	17.46 (1.31)	at 6 mo	17.22 (1.40)	17.09^*^ (1.35)	17.21 (1.38)
at 18 mo	16.51 (1.20)	16.53 (1.20)	16.39^*^ (1.12)	at 18 mo	16.16 (1.22)	16.16 (1.20)	16.08 (1.18)
at 42 mo	15.82 (1.16)	15.78 (1.15)	15.71^*^ (1.09)	at 42 mo	15.69 (1.22)	15.65 (1.20)	15.60 (1.25)
**Weight, g**				**Weight, g**			
at 3 mo	6,942 (881)	6,879 (810)	6996 (833)	at 3 mo	6,518 (785)	6,400^*^ (784)	6,470 (784)
at 6 mo	8,960 (936)	8,955 (931)	8,982 (927)	at 6 mo	8,498 (904)	8,426 (883)	8,453 (902)
at 18 mo	10,821 (1,083)	10,799 (1,052)	10,737 (1,091)	at 18 mo	10,264 (1,024)	10,189 (1,029)	10,160^*^ (1,005)
at 42 mo	14,564 (1,536)	14,624 (1,567)	14,546 (1,577)	at 42 mo	14,196 (1,566)	14,125 (1,559)	14,069 (1,609)
**Height, cm**				**Height, cm**			
at 3 mo	62.9 (2.8)	62.7 (2.6)	63.2^*^ (2.6)	at 3 mo	61.8 (2.5)	61.2^*^ (2.5)	61.5^*^ (2.5)
at 6 mo	71.5 (2.6)	71.7 (2.6)	71.7 (2.6)	at 6 mo	70.2 (2.5)	70.2 (2.5)	70.0 (2.5)
at 18 mo	80.9 (2.7)	80.8 (2.7)	80.9 (2.8)	at 18 mo	79.7 (2.6)	79.4^*^ (2.7)	79.4 (2.7)
at 42 mo	95.9 (3.4)	96.2^*^ (3.5)	96.1 (3.6)	at 42 mo	95.0 (3.4)	94.9 (3.4)	94.9 (3.5)

BMI, body mass index.

Data show the means (standard deviation). ^*^ indicates statistical significance at a *P* level <0.05 vs. the unaffected group.

### Changes in the BMI, weight, and height following the earthquake

**Figure 1.**
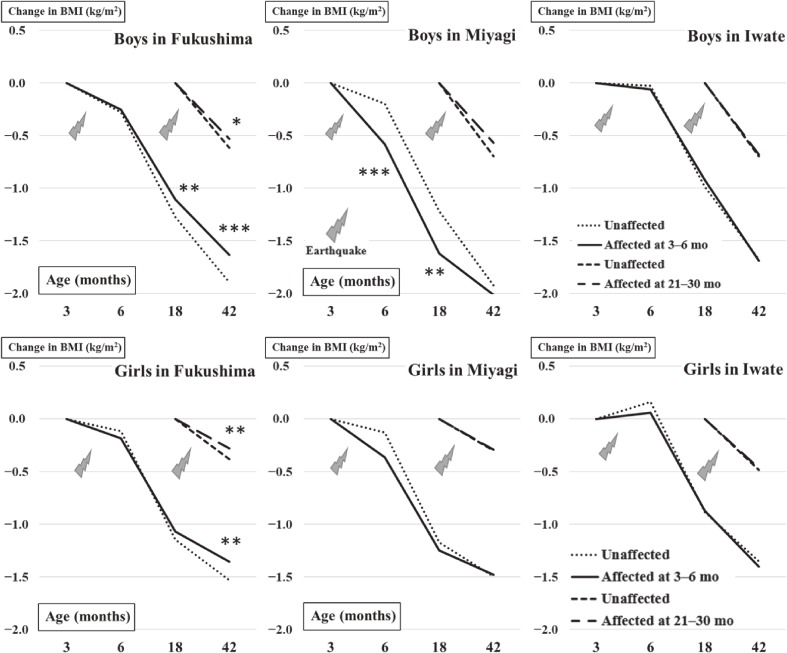
Change in BMI with adjustment of the gestational age in affected and unaffected infants after the Great East Japan Earthquake. ^*^P < 0.05, ^**^P < 0.01 and ^***^P < 0.001 at 6, 18 and 42 months old in infants affected at an age of 3 to 6 months vs. the unaffected infants; Bonferroni's method for multiple comparisons.

Table 3 shows the differences between affected and unaffected groups in adjusted changes in the BMI, weight, and height from baseline to 42 months of age. Figure 1 illustrates the changes in BMI in the two affected groups and those unaffected with an adjustment for the gestational age. In the group affected between 3 and 6 months old in Fukushima, there were significant increases in the changes in BMI from baseline for the boys at 18 (*P* = 0.0006) and 42 months (*P* < 0.0001) of age and for the girls at 42 months of age (*P* = 0.0005) compared with the unaffected group. In the group affected between 21 and 30 months old in Fukushima, there were significant increases in the BMI from baseline for both boys (*P* = 0.014) and girls (*P* = 0.0034) at 42 months old, compared with the unaffected group. In the group affected between 3 and 6 months old in Miyagi, there were significant decreases in the changes in BMI for the boys at 6 (*P* < 0.0001) and 18 months old (*P* = 0.0005) compared with the unaffected group. In the group affected between 21 and 30 months old in Miyagi, there was no significant change in the BMI of either boys or girls compared with the unaffected group. In the groups affected between 3 and 6 months old and 21 and 30 months old in Iwate, there were no significant changes in the BMI of either boys or girls compared with the unaffected group. These results were consistent with analyses of the changes in BMI in similar models with adjustment for birth weight and BMI at the 3-month health exam (eFigure 1 and eFigure 2).

**Table 3.** Difference in the adjusted changes in BMI, weight, and height in children affected at 3–6 and 21–30 months of age vs. unaffected children after the Great East Japan Earthquake

**Boys in Fukushima**	**Affected at 3–6 mo**	**Affected at 21–30 mo**	**Girls in Fukushima**	**Affected at 3–6 mo**	**Affected at 21–30 mo**
**BMI, kg/m^2^**			**BMI, kg/m^2^**		
at 3 mo	0		at 3 mo	0	
at 6 mo	+0.02		at 6 mo	−0.07	
at 18 mo	+0.16^**^	0	at 18 mo	+0.08	0
at 42 mo	+0.26^***^	+0.09	at 42 mo	+0.17^**^	+0.11^†^
**Weight, g**			**Weight, g**		
at 3 mo	0		at 3 mo	0	
at 6 mo	+27^*^		at 6 mo	+46	
at 18 mo	+24	0	at 18 mo	+37	0
at 42 mo	+88	−11	at 42 mo	+106	−14
**Height, cm**			**Height, cm**		
at 3 mo	0		at 3 mo	0	
at 6 mo	+0.1		at 6 mo	+0.4	
at 18 mo	−0.2	0	at 18 mo	+0.0	0
at 42 mo	−0.2	−0.2^†††^	at 42 mo	−0.0	−0.3^††^

**Boys in Miyagi**	**Affected at 3–6 mo**	**Affected at 21–30 mo**	**Girls in Miyagi**	**Affected at 3–6 mo**	**Affected at 21–30 mo**
**BMI, kg/m^2^**			**BMI, kg/m^2^**		
at 3 mo	0		at 3 mo	0	
at 6 mo	−0.38^***^		at 6 mo	−0.23	
at 18 mo	−0.40	0	at 18 mo	−0.08	0
at 42 mo	−0.09	+0.13	at 42 mo	+0.02	+0.01
**Weight, g**			**Weight, g**		
at 3 mo	0		at 3 mo	0	
at 6 mo	−33		at 6 mo	+80	
at 18 mo	−19	0	at 18 mo	+79	0
at 42 mo	+98	+3	at 42 mo	+179	+64
**Height, cm**			**Height, cm**		
at 3 mo	0		at 3 mo	0	
at 6 mo	+0.7		at 6 mo	+1.0	
at 18 mo	+0.8	0	at 18 mo	+0.5	0
at 42 mo	+0.4	−0.2^†††^	at 42 mo	+0.6	+0.1

**Boys in Iwate**	**Affected at 3–6 mo**	**Affected at 21–30 mo**	**Girls in Iwate**	**Affected at 3–6 mo**	**Affected at 21–30 mo**
**BMI, kg/m^2^**			**BMI, kg/m^2^**		
at 3 mo	0		at 3 mo	0	
at 6 mo	−0.04		at 6 mo	−0.10	
at 18 mo	+0.07	0	at 18 mo	+0.01	0
at 42 mo	−0.01	+0.02	at 42 mo	−0.05	+0.01
**Weight, g**			**Weight, g**		
at 3 mo	0		at 3 mo	0	
at 6 mo	+32		at 6 mo	0	
at 18 mo	−16	0	at 18 mo	−56	0
at 42 mo	+56	+35	at 42 mo	−68	−40
**Height, cm**			**Height, cm**		
at 3 mo	0		at 3 mo	0	
at 6 mo	+0.2		at 6 mo	+0.3	
at 18 mo	−0.2	0	at 18 mo	−0.2	0
at 42 mo	+0.3	+0.2	at 42 mo	+0.1	−0.1

BMI, body mass index.

^*^P < 0.05, ^**^P < 0.01 and ^***^P < 0.001 in infants affected at 3–6 months vs. unaffected infants after adjusting for multiple comparisons.

^†^*P* < 0.05, ^††^*P* < 0.01 and ^†††^*P* < 0.001 in children affected at 21–30 months vs. unaffected infants.

## DISCUSSION

### Interpretation within the context of previous studies

The results of the group affected between their 3- and 6-month health exams are in line with those of children in nursery and primary schools in northeast Japan.^8^ Our data on infants, toddlers, and preschool- and school-aged children indicate that in Fukushima, the increase in mean BMI was prolonged. A survey conducted in a large sample of adults between the ages of 40 and 90 years in Fukushima found mean BMI increases of +0.2 kg/m^2^ in non-evacuees and +0.6 kg/m^2^ in evacuees over an average follow-up period of 1.6 years.^22^ Increased sedentary behavior was seen among American elementary-school children after Hurricane Ike,^23^ and the same may have happened here. Inactivity could partially account for the prolonged increase in mean BMI in Fukushima.^24^

### Possible explanations

There are a number of possible reasons for the significant decrease in mean BMI of boys in Miyagi at 6 months old compared with the unaffected group (Figure 1). Small to intermediate aftershocks caused fear among the residents. Mothers with infants and young children were preoccupied by their own survival, health, housing, and economic problems and those of relatives. Stressful conditions are known to decrease the production of breast milk.^25^ Mothers may also have been concerned that their breast milk could become radioactive as a result of their diet,^26^^,^^27^ although such concerns are unfounded.^28^ It is likely that after the disaster, infants did not have an adequate intake of breast milk. Immediately after the earthquake, infant formula was also in short supply because of railway and highway damage.^13^ In response to donations by Japanese milk companies,^29^ the Fishery Agency of Japan and helicopters of the Self Defense Force delivered 43,100 kg of milk formula to evacuated mothers.^30^ Despite these efforts, there were shortages of milk formula in the face of increasing needs.^31^ Damaged water pipes and gas and electric lines^32^ made it difficult for mothers to wash and boil milk bottles and to dissolve milk formula in boiling water. Collectively, conditions in the wake of the earthquake may have disrupted the breast- and formula-feeding that infants require for normal growth.

The increased mean BMI at 42 months observed in the affected infants in Fukushima (Figure 1) might be explained by stress contributing to hormonal changes. Such changes include sleeplessness caused by a lack of outdoor play for fear of exposure to radiation, disruption of daily life caused by evacuation to provisional shelters or other residential housing, transmission of maternal frustration, and a depressed mood among residents. Increased peripheral adrenocorticotropic hormone, corticosterone, and catecholamine under stress could consume extra energy.^33^ Following the 1999 Taiwan earthquake, hyperleptinemia was reported in local residents, related to hyperarousal.^15^ In the post-quake phase in Japan, leptin, an appetite-regulating hormone,^34^ may have disrupted the eating behavior of the infants. The fear of radiation and an instinctive desire for survival might also have increased appetite.

There was a significantly positive difference in the changes in mean BMI of toddlers affected at 21 to 30 months old compared with unaffected toddlers in Fukushima. The specific reasons for this are unclear, but on average, the change in BMI in the toddler group in Fukushima was larger and the changes in height and weight were smaller than in the unaffected group. This finding is indicative of growth disturbances in a subset of the toddlers and may presage early adiposity rebounds and subsequent obesity.^35^ This phenomenon may also reflect a growth environment related to disaster stress- and diet-related problems.

In Miyagi and Iwate, the changes in the mean BMIs were different from Fukushima. The trend of increased BMI among infants and toddlers in Fukushima and decreased BMI among infants in Miyagi parallel the data obtained from nursery school children in a previous study.^8^ This observation may be attributable to different situations involving the total amount of physical activity and food supply in the three affected prefectures. A previous report has shown that the railways and educational environments of primary and secondary schools have recovered at different rates, fastest in Miyagi and slowest in Fukushima among the three prefectures from March 2011 to March 2013.^36^ The report described the rehabilitation scores for local infrastructure to be 81.1% in Fukushima, 91.0% in Miyagi and 88.0% in Iwate in March 2013, with a score of 100% representing pre-quake conditions. In Fukushima after the earthquake, the outdoor play of children was limited by their parents’ fear of radioactivity.^37^ A normal living environment and commercial logistics appear to have been restored earlier in Miyagi, perhaps in its role as the center of the northeast district of Japan. Although the sample size of children in Miyagi was relatively small, these situations may have caused different trajectories for the observed changes in mean BMI. The children in Iwate, the most distant prefecture from the nuclear power plant, could have re-established normal outdoor play and the consumption of local food early in the post-quake phase.

### Limitations

We should consider several limitations when interpreting the results. First, the sample population may not be representative of infants and toddlers in northeast Japan; the percentages of children in each prefecture who participated in the study (approximately 2.6–15.2%) were relatively small. Since a subset of residents left the prefectures, the accurate proportion of children in each prefecture is unknown. However, although the anthropometric data did not include severely-affected infants who moved away or died, a recent analysis suggests that the influence of those who moved out and died might be ignored.^24^ Second, measured height can vary, depending on the situation of the child health exam. Commonly, height is measured in the supine position at the 3- and 6-month exams, and in a standing position at 18 and 42 months. Weight can also fluctuate, depending upon the timing of meals. Third, adjustment for confounding factors may be insufficient. Maternal age, parity, and pregnancy complications could bias child growth. The data from the child health exams did not include these potential confounding factors. Finally, the time point of 42 months old might have been too late. We considered that the impact of the earthquake should be felt by infants and toddlers as they are at the beginning of physical growth and motor development. Since it was not possible to predict the timing of the impact, we used the maximum available data to evaluate the changes in BMI.

### Practical implications

In infancy, a decrease in BMI indicates a more rapid increase in height than weight.^38^ In normally-growing infants, the growth in height catches up with the increase in weight. Particularly in Fukushima, the infants in the group affected between 3 and 6 months old exhibited unbalanced changes in mean weight and height (Table 3). It appears that, at a population level, the trajectories of BMI in the infants begin to reach the minimum sooner than those in the unaffected group. The toddlers in Fukushima also weighed less and were shorter on average than those in the unaffected group. Since these phenomena of anthropometrics in an individual child may be a warning of growth disturbance and early adiposity rebound, pediatricians should follow the future growth of the affected infants and toddlers. Evidence from pediatric endocrinology indicates that an early adiposity rebound is a risk factor for future obesity and cardiovascular disease.^39^^,^^40^ We recommend that health administrators and pediatricians should carefully follow the affected children in Fukushima to assess their growth, as well as to check for the development of potential cardiovascular risks from adolescence to adulthood.

### Conclusions

The infants affected at between 3 and 6 months old in Fukushima Prefecture showed an increase in mean BMI towards 42 months compared with the reference group, which suggests a risk of potential early adiposity rebound at a population level. The data also suggested the potential risk of this in toddlers affected between 21 and 30 months old in Fukushima, with a smaller increase in mean height and weight. In Miyagi Prefecture, the mean BMI of the male infants decreased immediately after the earthquake but recovered. The results underscore the role of pediatricians in following the growth of affected infants when disasters occur, to prevent obesity and its complications.

## APPENDIX A. SUPPLEMENTARY DATA

eFigure 1. Change in BMI with adjustment of the birth weight of affected and unaffected infants after the Great East Japan Earthquake. *p < 0.05, **p < 0.01 and ***p < 0.001 at 6, 18 and 42 months old in infants affected at an age of 3 to 6 months vs. the unaffected infants; Bonferroni’s method for multiple comparisons.

eFigure 2. Change in BMI with adjustment of BMI at the 3-month health examination in affected and unaffected infants after the Great East Japan Earthquake. *p < 0.05, **p < 0.01 and ***p < 0.001 at 6, 18 and 42 months old in infants affected at an age of 3 to 6 months vs. the unaffected infants; Bonferroni’s method for multiple comparisons.
