# Supplementary material for: Impact of the Great East Japan Earthquake on Body Mass Index, Weight, and Height of Infants and Toddlers: An Infant Survey
Source: J Epidemiol. 2018 May 5;28(5):237–44. doi: 10.2188/jea.JE20170006 (PMC5911674; doi:10.2188/jea.JE20170006)
Supplement: Supplementary file 1 [file je-28-237-s001.pdf]

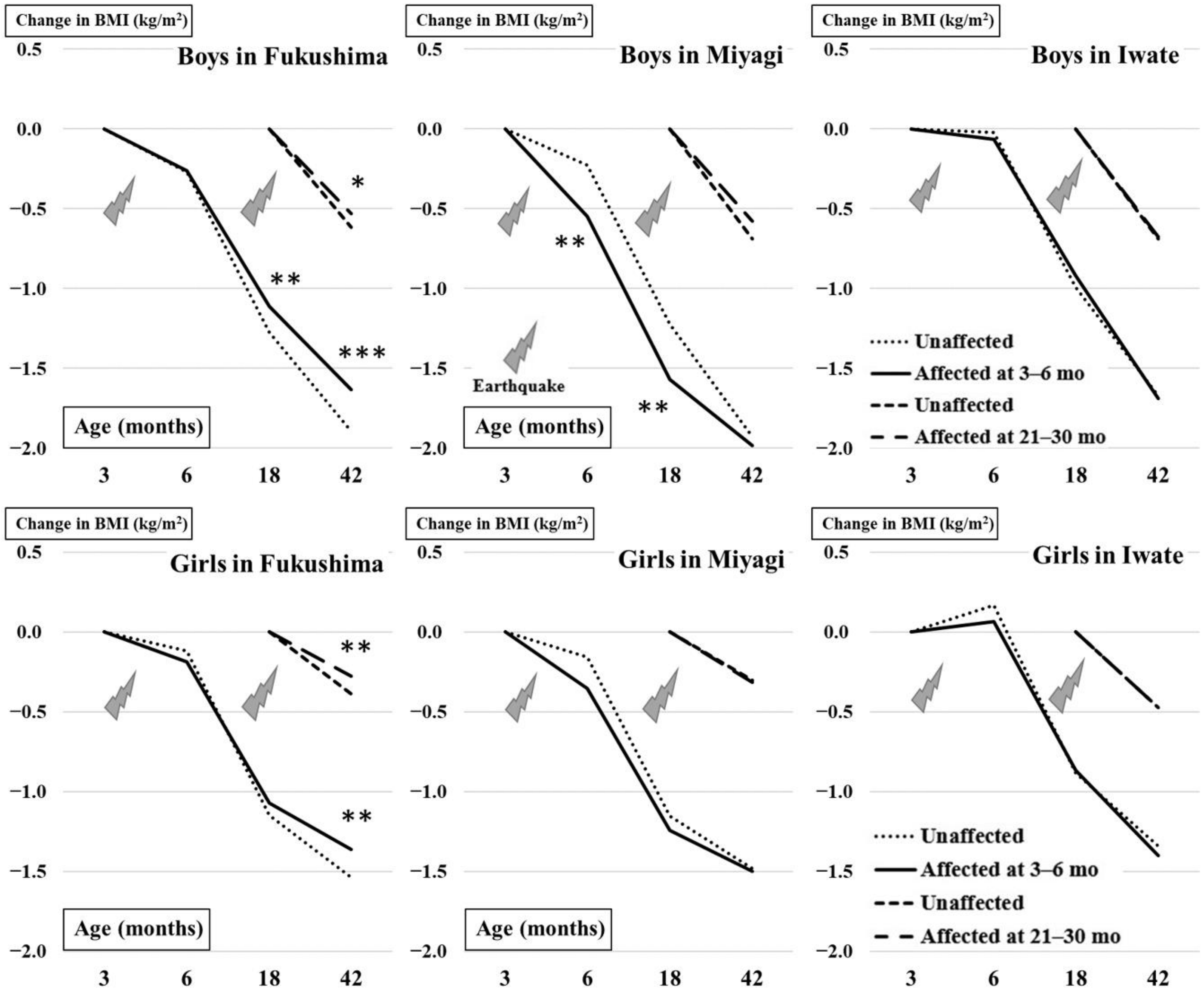

eFigure 1. Change in BMI with adjustment of the birth weight of affected and unaffected infants after the Great East Japan Earthquake. *$p < 0.05$, **$p < 0.01$ and ***$p < 0.001$ at 6, 18 and 42 months old in infants affected at an age of 3 to 6 months vs. the unaffected infants; Bonferroni's method for multiple comparisons.

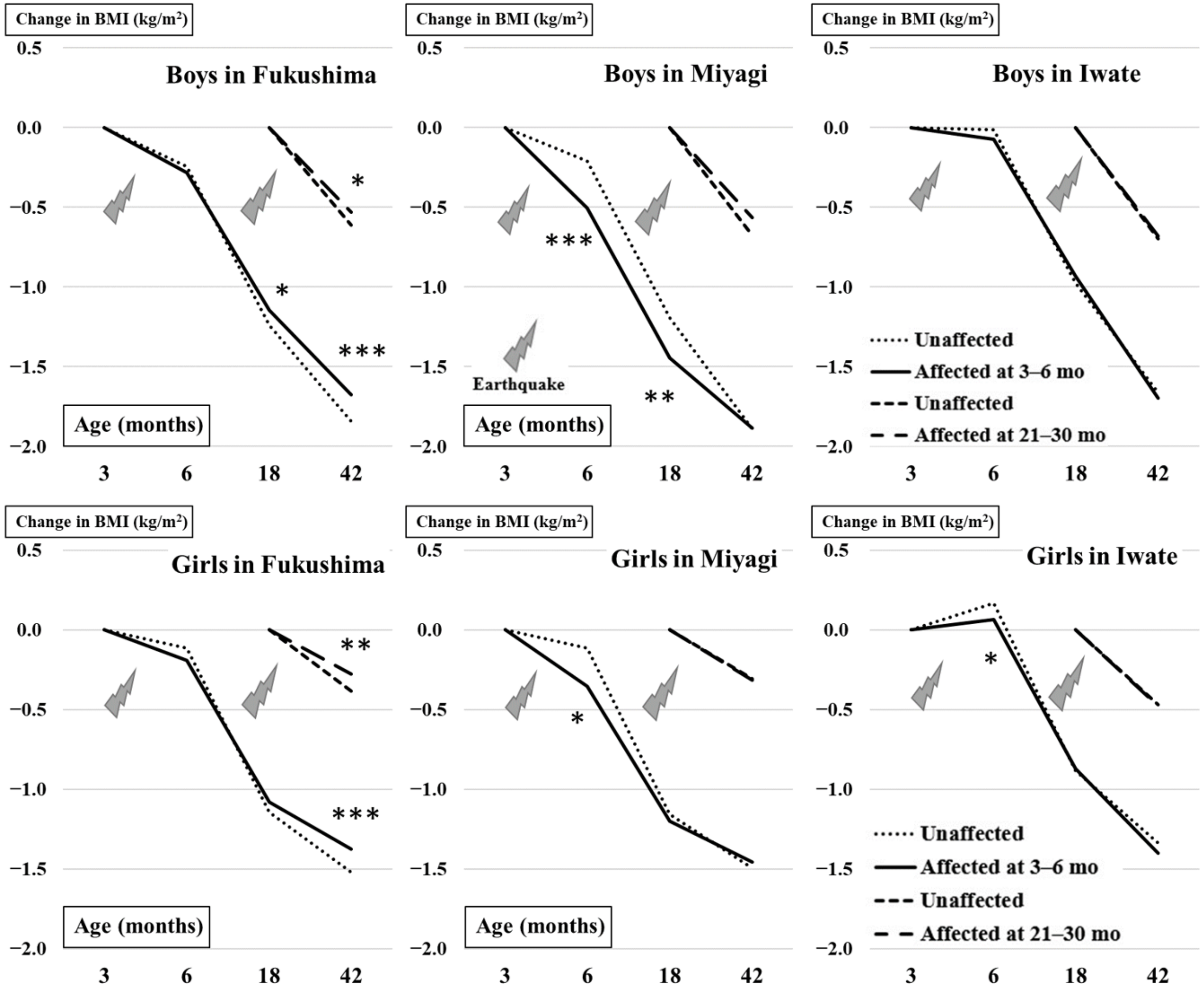

eFigure 2. Change in BMI with adjustment of BMI at the 3-month health examination in affected and unaffected infants after the Great East Japan Earthquake. *p < 0.05, **p < 0.01 and ***p < 0.001 at 6, 18 and 42 months old in infants affected at an age of 3 to 6 months vs. the unaffected infants; Bonferroni's method for multiple comparisons.
